# Supplementary material for: Airborne dispersion of droplets during coughing: a physical model of viral transmission
Source: Sci Rep. 2021 Feb 25;11:4617. doi: 10.1038/s41598-021-84245-2 (PMC7907382; doi:10.1038/s41598-021-84245-2)
Supplement: Supplementary file 1 — Supplementary Information [file 41598_2021_84245_MOESM1_ESM.docx]

**Supplementary Information**

**Airborne Dispersion of Droplets during Coughing: A Physical Model of Viral Transmission**

Hongying Li, PhD^1^, Fong Yew Leong, PhD^1†^, George Xu, PhD^1^, Chang Wei Kang, PhD^1^, Keng Hui Lim, PhD^1^, Ban Hock Tan, FRCP(UK)^2^, Chian Min Loo, MBBS^3^

^1^ A*STAR Institute of High Performance Computing, 1 Fusionopolis Way, Connexis, Singapore 138632

^2^ Department of Infectious Diseases, Singapore General Hospital, Outram Road, Singapore 169608

^3^ Department of Respiratory and Critical Care Medicine, Singapore General Hospital, Outram Road, Singapore 169608

**Cough droplet distribution and viral load**

The size distribution of droplets emitted in a cough is reproduced from a seminal study by Duguid^1^, as shown in Supplementary Table S1. More recent measurements, carried out using different methods and under different conditions, reported size distributions that are predominantly skewed towards larger droplets (50–100 µm)^2,3^. These distributions may not sufficiently represent smaller droplet sizes emitted during a cough.^4^

| **Diameter (μm)** | **2** | **4** | **8** | **16** | **24** | **32** | **40** | **50** |
| --- | --- | --- | --- | --- | --- | --- | --- | --- |
| Number count^*^ | 50 | 290 | 970 | 1,600 | 870 | 420 | 240 | 110 |
| Median viral count (copies/droplet)^†^ | 1.38×10^-5^ | 1.11×10^-4^ | 8.84×10^-4^ | 7.08×10^-3^ | 2.39×10^-2^ | 5.66×10^-2^ | 0.111 | 0.215 |
| Day-0 viral count (copies/droplet)^‡^ | 2.38×10^-4^ | 1.92×10^-3^ | 1.53×10^-2^ | 0.122 | 0.413 | 0.978 | 1.917 | 3.714 |
|  |  |  |  |  |  |  |  |  |
| **Diameter (μm)** | **75** | **100** | **125** | **150** | **200** | **250** | **500** | **1000** |
| Number count^*^ | 140 | 85 | 48 | 38 | 35 | 29 | 34 | - |
| Median viral count (copies/droplet)^†^ | 0.729 | 1.728 | 3.375 | 5.832 | 13.82 | 27.00 | 216.0 | - |
| Day-0 viral count (copies/droplet)^‡^ | 12.59 | 29.84 | 58.30 | 100.7 | 238.7 | 466.4 | 3,731 | - |
| ^*^ Reproduced from Duguid.^1^  ^†^ Based on median SARS-CoV-2 viral loading of 3.3×10^6^ copies/mL.^2^  ^‡^ Based on SARS-CoV-2 viral loading from hospitalized patients on day of admission of 5.7×10^7^ copies/mL.^2^ | | | | | | | | |

**Supplementary Table S1.** Droplet size distribution and SARS-CoV-2 viral load in a typical cough.

Based on saliva samples of hospitalized patients, the median SARS-CoV-2 viral load is found to be 3.3×10^6^ copies/mL, with a range from 9.2×10^2^ to 2.0×10^8^ copies/mL.^5^ Day-0 viral loading from admission day samples was reported to be 5.7×10^7^ copies/mL and this sets the transmission risk limit for our modelled source. Assuming that salivary viral loading rates are conserved in droplets emitted during a cough, the viral content in a droplet can be determined depending on its size. Droplets with diameters greater than 100 µm neglected in this study due to their extremely fast settling rates and low dispersion potential.

For modelling purposes, droplets are generated based on discrete size distributions and injected randomly within mouth region at the start of the simulation.

**Governing equations**

The problem mentioned above is governed by fluid flow, droplet movement as well droplet evaporation for the evaporation scenario. The governing equations then include Navier-Stokes equation, proper turbulent flow model as well as discrete phase movement and droplet evaporation equations, respectively.

The governing equations for fluid mass and momentum with turbulence are

,

(S1)

,

(S2)

where κ is the turbulent kinetic energy and ε is the dissipation of turbulent energy, expressed as

,

(S3)

,

(S4)

where C_1ε_ and C_2ε_ are constants 1.44 and 1.92 respectively, σ_κ_ and σ_ε_ are 1.00 and 1.3 respectively^8^ and G_k_ is the production of turbulence kinetic energy.

Eddy viscosity µ_τ_ is expressed as

$\mu_{t}=\rho C_{\mu}\frac{\kappa^{2}}{\varepsilon}$,

(S5)

where *C_µ_* is equal to 0.09.

The source terms in continuity and momentum (Equations S1 and S2) accounts for fluid loss via evaporation,

,

(S6)

,

(S7)

where $m_{d0}$is the mass of droplet, is the rate of change of droplet mass and *V* is the control volume.

In addition to solving the flow field, species transport equations are also solved. Air is assumed to consist three main species components, i.e. O_2_, N_2_ and H_2_O vapor. The mass fraction of O_2_ and H_2_O is solved by

,

(S8)

where $\vec{J}_{i}$ is the diffusive flux of species i and can be expressed as,

$\vec{J}_{i}=-\left(\rho D_{i,m}+\frac{\mu_{t}}{Sc_{t}}\right)\nabla x_{i}-D_{t}\frac{\nabla T}{T}$,

(S9)

where Sc_t_ is the turbulent Schmidt number (taken as 0.7) and D_t_ is the turbulence diffusivity. The source term of species i is simply

$S_{i}=\frac{dm_{d}}{dt}\frac{1}{V}$.

(S10)

The energy conservation equation is,

,

(S11)

where E is energy,

,

(S12)

h is the sensible heat,

$h=\sum_{i} x_{i}C_{p,i}T+\frac{P}{\rho}$,

(S13)

S_h_ is thermal source term,

,

(S14)

where the subscripts in and out identifies droplets entering and exiting a control volume.

The equation of motion of a droplet (subscript d) is

,

(S15)

$\vec{u}_{d}$ and $\vec{u}$ are the droplet and air velocities respectively. F_D_ is the drag force,

$F_{D}=\frac{18\mu}{\rho_{d}D_{d}^{2}}+\frac{C_{D}\mathrm{Re}}{24}$,

(S16)

where D_d_ is the droplet diameter and C_d_ is the drag coefficient (Morsi and Alexander, 1972) as a function of the droplet Reynolds number,^7^

$C_{D}=c_{1}+\frac{c_{2}}{\mathrm{Re}}+\frac{c_{3}}{\mathrm{Re}^{2}}$,

(S17)

,

(S18)

where c_1_, c_1_, c_3_ are empirical constants for spherical droplets estimates at following Reynolds number intervals,

$$c_{1},c_{2},c_{3}=\left\{\begin{matrix} 0, & 24, & 0 & & 0<\mathrm{Re}<0.1 \\ 3.69, & 22.73, & 0.0903 & & 0.1<\mathrm{Re}<1 \\ 1.222, & 1.667, & -3.889 & & 1<\mathrm{Re}<10 \\ 0.6167, & 46.5, & -116.67 & & 10<\mathrm{Re}<100 \\ 0.3644, & 98.33, & -2778 & & 100<\mathrm{Re}<1000 \\ 0.357, & 148.62, & -47500 & & 1000<\mathrm{Re}<5000 \\ 0.46, & -490.546, & 578700 & & 5000<\mathrm{Re}<10000 \\ 0.5191, & -1662.5, & 5416700 & & \mathrm{Re}>10000 \end{matrix}\right.$$

(S19)

Droplet evaporation is governed by diffusive flux of droplet vapor into the air,

$N_{v}=k_{c}\left(C_{vd}-C_{va}\right)$,

(S20)

where N_v_ is the molar evaporative flux of vapor and k_c_ is the mass transfer coefficient. C_vd_ is the saturated vapor pressure P_sat_ at the droplet surface,

$C_{vd}=\frac{P_{sat}}{RT_{d}}$ ,

(S21)

where T_d_ is droplet surface temperature. C_va_ is the partial vapor pressure,

$C_{va}=x_{v}\frac{P}{RT_{a}}$,

(S22)

where x_v_ is species mole fraction, P and T_a_ are the local pressure and temperature respectively. The mass transfer coefficient k_c_ is correlated with Reynolds number and Schmidt number,^8^

$k_{c}=\frac{D_{dv}}{D_{d}}\left(2.0+0.6\mathrm{Re}^{0.5}Sc^{1/3}\right)$,

(S23)

where D_dv_ is the diffusion coefficient of vapor in the air. The mass of the droplet evolves as

$m_{d}\left(t+\Delta t\right)=m_{d}\left(t\right)-N_{v}A_{d}M_{d}\Delta t$,

(S24)

where m_d_ is the droplet mass, M_d_ the molecular weight and A_d_ is the surface area.

The droplet temperature is governed by thermal balance including latent and sensible heats,

$$m_{d}C_{pd}\frac{dT_{d}}{dt}=hA_{d}\left(T_{a}-T_{d}\right)-\frac{dm_{d}}{dt}h_{fg}$$

(S25)

where h_fg_ is the latent heat of droplet. The convection heat transfer coefficient h is calculated with a modified Nusselt number,^9^

$h=\frac{\lambda\ln\left(1+B{}_{T}\right)}{D_{d}B_{T}}\left(2+0.6\mathrm{Re}_{d}^{0.5}\Pr^{\frac{1}{3}}\right)$,

(S26)

where Pr is the Prandtl number and λ is the thermal conductivity of air. B_T_ is the Spalding heat transfer number,

,

(S27)

where is the droplet evaporation rate and q_d_ is the heat energy transferred to the droplet.

**Evaporation model**

The evaporation model is verified in independent tests on 1, 10 and 100 µm droplets under relative humidity levels 0, 0.6 and 0.8 (Supplementary Figure S1). At relative humidity 0.6, a 10 µm droplet evaporates completely in 0.2 s, and a 100 µm in 2 s, which is comparable to simulation time scales, so evaporation is clearly an important factor in droplet dispersion. We compare our evaporation model against other studies and find agreement on droplet evaporation times across droplet sizes, based on Reynolds numbers, humidity and temperature gradients.^10,11^


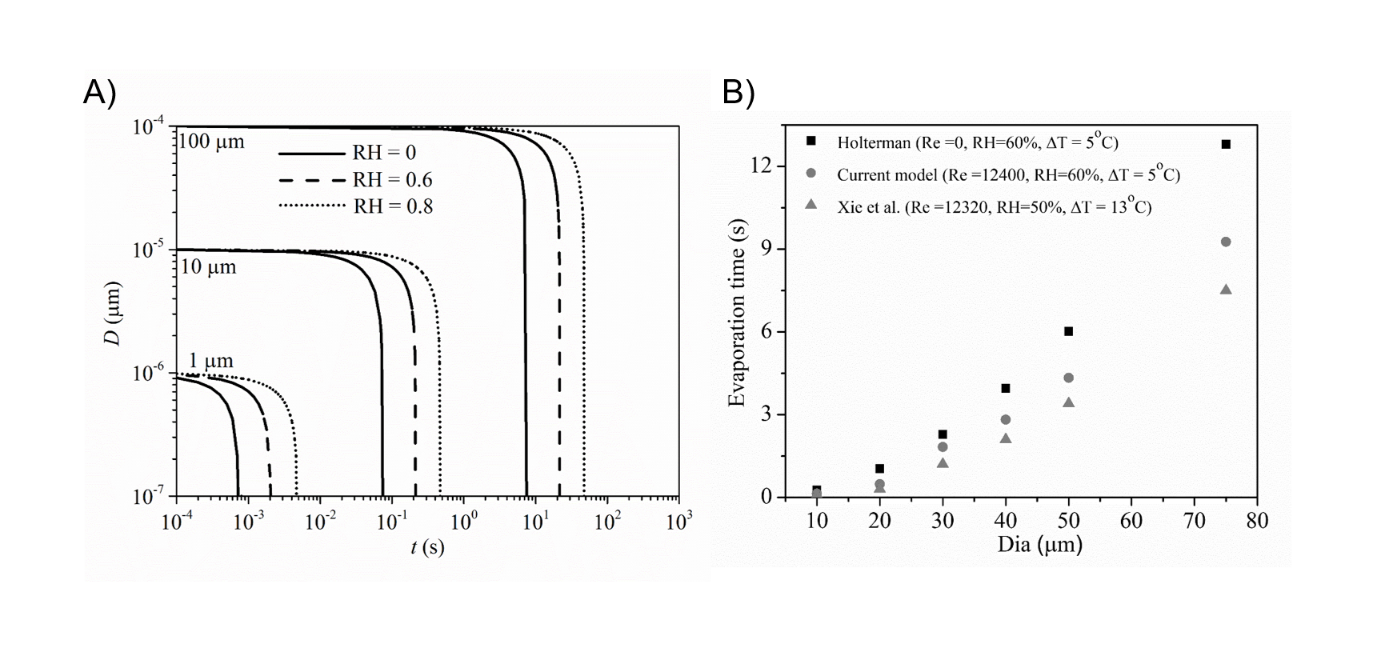


**Supplementary Figure S1.** A) Evaporation time for a droplet of diameter 1, 10 and 100 µm under relative humidity levels 0, 0.6 and 0.8. B) Evaporation times generated by present model compare well with those reported by other references.^10,11^

**Computational details**

The simulation domain is a rectangular box with dimensions of 10·1 m (length) × 7·12 m (width) × 5·4 m (height). Computational meshing is conducted directly on ANSYS FLUENT (2019R3) using polyhedral unstructured scheme. The human model is geometrically meshed as a static obstacle 1·7 m with open arms. Additional mesh refinement is implemented using double boundary layers near geometric surface of the human models. The number of mesh elements is approximately 3·9 million.

Simulations are run on standalone workstation with 88 hyper-threaded CPUs and 384G RAM. Each job is assigned 12 CPUs and 64G RAM and resolves 30 s of simulation time in approximately 24 h real time.

**References**

1. Duguid, J.P. The size and the duration of air-carriage of respiratory droplets and droplet-nuclei. *Epidemiology & Infection* **44(6)**, 471-479 (1946).
2. Xie, X., Li, Y., Sun, H. & Liu, L. Exhaled droplets due to talking and coughing. *J. R. Soc. Interface* **6**, S703-714 (2009).
3. Loudon, R.G. & Roberts, R.M. Droplet expulsion from the respiratory tract. Am. Rev. Resp. Dis. **95**, 435-442 (1967).
4. Bourouiba L, Dehandschoewercker E, Bush JW. Violent expiratory events: on coughing and sneezing. *Journal of Fluid Mechanics* **745**, 537-563 (2014).
5. To, K.K. *et al.* Consistent detection of 2019 novel coronavirus in saliva. *Clinical Infectious Diseases* ciaa149 (2020). doi:10.1093/cid/ciaa149
6. Cundall, P.A. & Strack, O.D. A discrete numerical model for granular assemblies. *geotechnique* **29(1)**, 47-65 (1979).
7. Morsi, S.A. and Alexander, A.J. An investigation of particle trajectories in two-phase flow systems. *Journal of Fluid mechanics* **55(2)**, 193-208 (1972).
8. Ranz, W.E. & Marshall, W.R. Evaporation from drops. *Chem. eng. prog.* **48(3)**, 141-146 (1952).
9. Sazhin, S.S. Advanced models of fuel droplet heating and evaporation. *Progress in energy and combustion science* **32(2)**, 162-214 (2006).
10. Holterman, H.J. Kinetics and evaporation of water drops in air. (Wageninger: IMAG; 2003 Jul).
11. Xie X, Li Y, Chwang AT, Ho PL, Seto WH. How far droplets can move in indoor environments: revisiting the Wells evaporation-falling curve. *Indoor air* **17(3)**, 211-225 (2007).
